# Association Between Cognitive Impairment and Dysphagia: A Two‐Sample Mendelian Randomization Study

**DOI:** 10.1002/brb3.70295

**Published:** 2025-02-09

**Authors:** Yueqin Tian, Jiahui Hu, Qianqian Wang, Jia Qiao, Hongmei Wen, Qiuping Ye, Zulin Dou

**Affiliations:** ^1^ Department of Rehabilitation Medicine The Third Affiliated Hospital, Sun Yat‐sen University Guangzhou China; ^2^ Clinical Medical College of Acu‐Moxi and Rehabilitation Guangzhou University of Chinese Medicine Guangzhou China

**Keywords:** causality, cognitive impairment, dysphagia, Mendelian randomization

## Abstract

**Introduction:**

Previous observational studies have implied a correlation between cognitive impairment and dysphagia, but some have indicated no correlation between the two. Such contradictory findings may have been influenced by small sample sizes and potential confounders. In this Mendelian randomization (MR) analysis, we genetically estimated a causal relationship between cognitive impairment and dysphagia.

**Methods:**

The study included a large meta‐analysis of genome‐wide association studies (GWAS) of cognitive impairment in 269,867 individuals of European ancestry and pooled data from a GWAS of dysphagia in 165,765 individuals of European ancestry (cases 3497, controls 161,968). We then used five different complementary MR methods, including IVW, MR‐Egger, MR‐RAPS, weighted median, and weighted mean, to estimate causality between cognitive impairment and dysphagia and finally also assessed heterogeneity and horizontal pleiotropy by extensive sensitivity tests.

**Results:**

No evidence of heterogeneity in the effect of instrumental variables was found in Cochran's *Q* test; therefore, a fixed effects model was used. IVW analysis (OR: 1.206, 95% CI: [1.041, 1.371], *p* = 0.00508) found that cognitive impairment was associated with an increased risk of dysphagia and that there was a causal association between the two. Also, the weighted median (OR: 1.248, 95% CI: [1.012, 1.484], *p* = 0.0253), weighted mode (OR: 1.216, 95% CI: [1.043, 1.389], *p* = 0.0412), and MR‐RAPS (OR: 1.225, 95% CI: [1.069, 1.381], *p* = 0.00627) validated the conclusions. Furthermore, extensive sensitivity analyses found no evidence of heterogeneity or horizontal pleiotropy, confirming the reliability of this MR result.

**Conclusion:**

Our MR study demonstrated a causal effect of cognitive impairment on dysphagia from a genetic perspective, suggesting that individuals with a history of cognitive impairment require specific clinical attention to prevent the development of dysphagia.

## Graphical abstract

Large‐scale GWAS and MR analysis link cognitive impairment to increased dysphagia risk, suggesting a genetic causal effect and clinical implications for prevention.

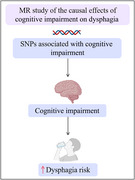

## 1 Introduction

Dysphagia is an abnormality in the passage of food from the mouth to the stomach that impairs the patient's safety in swallowing and eating, often leading to aspiration pneumonia, dehydration, malnutrition, reduced quality of life (Cichero et al. 2013; Roldan‐Vasco et al. 2021; van der Maarel‐Wierink et al. 2011), prolonged hospitalization, and increased mortality (Dell'Aquila et al. 2022). Dysphagia is often secondary to aging or multiple neurological disorders such as stroke, dementia, and Parkinson's disease (Airoldi et al. 2011; Kertscher et al. 2015; Rodrigues et al. 2011). Currently, risk factors are thought to include aging, low BMI, malnutrition, poor oral health, dehydration, cognitive impairment, and frailty (Leira et al. 2023). The incidence of dysphagia in the general adult population is estimated to be as high as 16.1% in the United States (Adkins et al. 2020). In the global population, about 8% of the population has been diagnosed with dysphagia, which means that about 590 million people around the world suffer from this disorder (Cichero et al. 2017). In the population over 60 years of age, this percentage rises between 10% and 33% (Thiyagalingam et al. 2021). Furthermore, the percentage of African populations with dysphagia is even higher at 64.2% (Dell'Aquila et al. 2022). With the aging of the global population, dysphagia will become an increasing public health problem. To better treat and alleviate the suffering of dysphagia patients, it is urgent to further understand the etiology and pathogenesis of dysphagia.

Cognitive functions primarily include memory, executive function, attention, language skills, and visuospatial abilities, and cognitive impairment usually refers to the impairment of at least one of these functions. Cognitive impairment can seriously affect the quality of life of patients and make them dependent on others for care. Similar to dysphagia, the prevalence of cognitive impairment increases with age (Cao et al. 2020; Hu et al. 2017; Petersen et al. 2018). It is estimated that up to 42% of the global population over 60 years old suffers from cognitive impairment (Ward et al. 2012). Dementia is a serious cognitive impairment that afflicts 1.8% of people in their 60s, 5.1% of people in their 70s, 15.1% of people in their 80s, and 35.7% of people in their 90s worldwide (Cao et al. 2020). Clinically, physicians have found that patients with dysphagia are often comorbid with cognitive impairment. Researchers speculate that cognitive decline affects the perception or intake of things during swallowing (Malandraki et al. 2009). Based on this conjecture, researchers have explored the relationship between cognitive impairment and dysphagia through many observational studies. A study on Japanese older adults dependent on long‐term care reported a negative correlation between the severity of cognitive impairment and swallowing function (Sakai et al. 2016). Another cross‐sectional study showed that nursing home older adults with lower cognitive functioning often had higher odds of swallowing disorder risk (Yatabe et al. 2018). Frontotemporal lobe damage may explain concomitant impairment of cognitive and swallowing functions. On the one hand, frontal lobe damage can result in executive dysfunction, characterized by a delayed oral phase, hesitation in swallowing, and momentary tongue immobility, affecting complex swallowing movements (Moon et al. 2018; Saito et al. 2016). Additionally, temporal lobe damage may impair language comprehension, perception, and recognition of food, indirectly impacting swallowing as these faculties play a pivotal role in food intake and the orchestration of swallowing actions (Coppin 2016; Defenderfer et al. 2017). On the other hand, temporal lobe damage can impact memory, language, and visual recognition—key elements of cognitive performance (Berron et al. 2018; Collin, Milivojevic, and Doeller 2015), while frontal lobe damage often results in executive dysfunction, which affects planning, decision‐making, and problem‐solving abilities (Cristofori, Cohen‐Zimerman, and Grafman 2019). Frontotemporal dementia (FTD), characterized by atrophy of the frontal and temporal lobes, often leads to challenges with language expression or naming, eating, and swallowing (Bang, Spina, and Miller 2015; Lewis et al. 2018).

However, it is worth noting that some earlier studies reported no association between cognitive impairment and dysphagia (Holland et al. 2011; Maeda et al. 2018; Michel et al. 2018). One such study of community‐based patients with dementia found that there was no significant relationship between the severity of oropharyngeal dysphagia and cognitive impairment (Michel et al. 2018). In summary, current research is unable to determine the association and exact direction between cognitive impairment and dysphagia. To learn more about the interactions between cognitive impairment and dysphagia and to put forward clinical strategies for diagnosis and treatment to help the comorbid population with both disorders, it is imperative to identify the underlying causality between them.

Limited by study design, observational studies have some inherent disadvantages, including reverse causality, measurement error, and potential bias. Mendelian randomization (MR), which is based on pooled statistics from large‐scale genome‐wide association studies (GWASs), can overcome these biases by making causal inferences from the perspective of gene prediction and is now widely used in the field of epidemiological pathogenesis (Bowden and Holmes 2019; Lawlor et al. 2008). To obtain fair and objective results, MR must fulfill three assumptions (Davies et al. 2018; Emdin, Khera, and Kathiresan 2017): first, the instrumental variables (IVs) are directly and significantly associated with exposure; second, the IVs are independent of outcome‐related confounders; and third, the IVs influence the outcome through only one pathway, exposure. In this study, based on the pooled statistics of cognitive impairment and dysphagia from a large‐scale GWAS, we performed a two‐sample MR analysis to reveal the causality between cognitive impairment and dysphagia. According to the three hypotheses of MR, if cognitive impairment causally affects dysphagia, then variants affecting cognitive impairment should, to some extent, also affect dysphagia. However, horizontal pleiotropy implies that the variant could affect dysphagia by other means and should be excluded first.

## 2 Methods

### 2.1 Study Design

**FIGURE 1:**
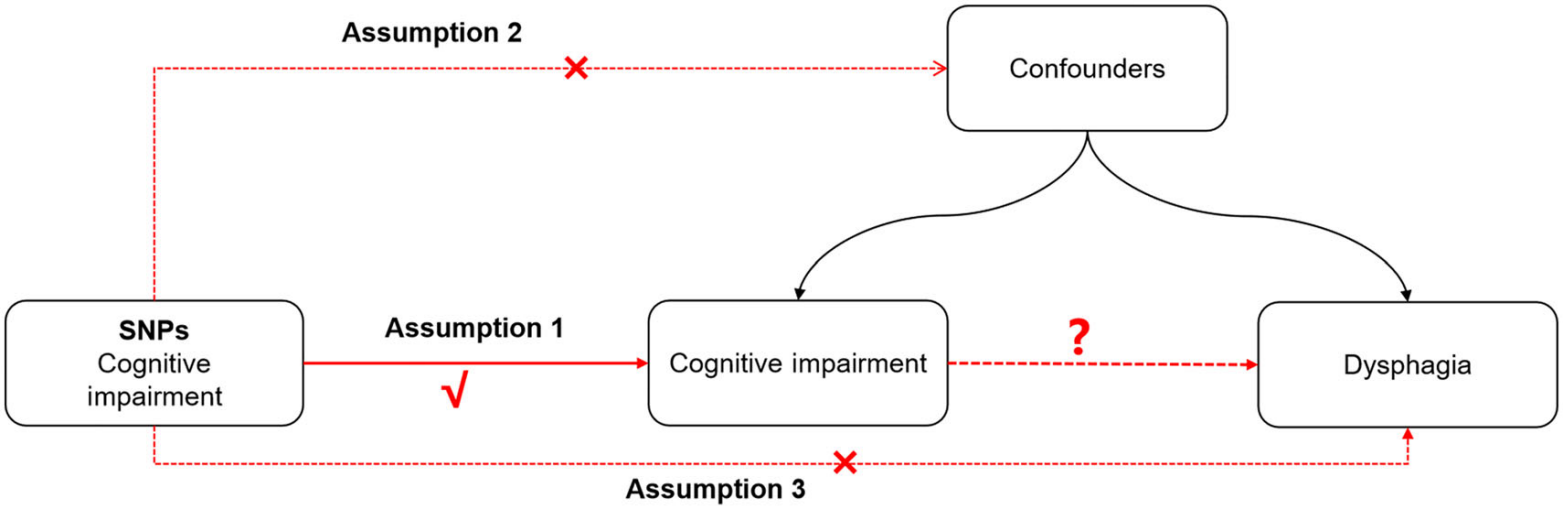
The principles of MR analysis.

Two‐sample MR analyses explored the causality between exposure and outcome with significant genetic variation of exposure as an IV. The working principle of this study is shown in Figure 1. To exclude possible bias due to demographics, we limited the MR study population to individuals with a genetic background of European ancestry.

The primary genetic instruments were derived from pooled data from publicly available GWAS databases. GWAS summary data for dysphagia was extracted from the FinnGen Consortium (GWAS ID: finn‐b‐R18_DYSPHAGIA, https://gwas.mrcieu.ac.uk/datasets/finn‐b‐R18_DYSPHAGIA/). The dataset analyzed 165,465 Europeans (3497 dysphagia patients and 161,968 controls) and obtained 16,380,224 single nucleotide polymorphisms (SNPs). Genetic data on cognitive impairment were extracted from a GWAS meta‐analysis of 269,867 individuals of European ethnicity, which identified 205 genomic loci strongly associated with cognition (Savage et al. 2018). Notably, we utilized the online calculation tool (https://sb452.shinyapps.io/power) to perform sample size calculations (Burgess 2014). As the previous study stated (Sun et al. 2022), the sample size calculation relied on several parameters (Brion, Shakhbazov, and Visscher 2013), including the proportion of variance in the exposure variable explained by SNPs (*R* ^2^_xz_), true odds ratio (OR) of the outcome variable per standard deviation of the exposure variable, and statistical power and proportion of cases in the study (K). The calculation results showed that the sample size required to detect a causal association between cognitive impairment and dysphagia in this study was 154,720, which indicates that the sample size included in this study was adequate.

This research is a secondary analysis of public GWAS summary data. The original GWAS studies have all received ethical approval. Furthermore, this study did not use individual‐level data, so it does not require new ethics committee approval.

### 2.2 Selection of Genetic IVs

Based on the three main hypotheses of MR, we performed a series of quality controls to screen qualified genetic IVs. First, independent SNPs strongly associated with cognitive impairment (*p* < 5 × 10^−8^) were selected (Seyedsalehi et al. 2022). Second, SNPs strongly associated with dysphagia were screened by *p* < 5 × 10^−6^. Third, to avoid being affected by strong linkage disequilibrium, we chose SNPs that met *r* ^2^ < 0.001 and kb > 1000 (The 1000 Genomes Project Consortium 2010). Fourth, sample overlap effects and exclusion of weak instrumental bias were also assessed by calculating the *F*‐statistic, which was calculated as follows: *F* = *R* ^2^ × (*N−*2)/(1*−R* ^2^). *R* ^2^ denotes the exposure variance explained by each IV, and *N* denotes the sample size of the GWAS. Those IVs with *F*‐statistics below 10 were considered weak IVs and would be eliminated (Burgess, Thompson, and CRP CHD Genetics Collaboration 2011). Finally, if the target SNPs were unavailable in the final dataset, they were substituted with alternative SNPs that were in strong linkage disequilibrium with the target SNPs.

### 2.3 MR Analysis

In this study, to yield accurate and robust results, we utilized five different complementary methods to assess the causal effect of cognitive impairment on dysphagia. These methods included inverse variance weighting (IVW), MR‐Egger regression, weighted median, weighted mode, and MR robust adjusted profile score (MR‐RAPS). First, the IVW method is primarily used to estimate the underlying causality and provides the most accurate results, but it requires that the selected IVs must satisfy all three major assumptions of the MR analysis (Burgess, Butterworth, and Thompson 2013). Second, MR‐Egger performs a weighted linear regression that leaves its causal effect estimates unaffected despite the invalidity of the IVs (Bowden, Davey Smith, and Burgess 2015). Third, the weighted median approach computes the weighted median of the Wald ratio estimates, which reduces the bias caused by horizontal pleiotropy and is more robust (Bowden et al. 2016). Fourth, the weighted model approach adjusts for the effect of differences in genotype frequencies on the results by weighting different genotypes (Hartwig, Davey Smith, and Bowden 2017). Fifth, MR‐RAPS could correct the horizontal pleiotropy and thus reduce the bias (Zhao et al. 2019). The MR results are expressed as ORs and corresponding 95% confidence intervals (CIs).

### 2.4 Sensitivity Analysis

First, to quantify the heterogeneity of SNPs, we performed Cochran's *Q* test by MR‐Egger and IVW. A *p* value greater than 0.05 would indicate the absence of heterogeneity and that a fixed effects model would be used in subsequent MR analyses; otherwise, it would indicate the presence of heterogeneity and the need to use a random effects model (Burgess, Small, and Thompson 2017). Next, to detect the possibility of horizontal pleiotropy, we applied the MR‐Egger and MR multinomial residuals and outliers (MR‐PRESSO) tests. The intercept of MR‐Egger regression can represent the average multiplicity of IVs. A *p* value of the intercept greater than 0.05 suggests the absence of horizontal multiplicity; otherwise, it suggests the presence of horizontal multiplicity (Bowden, Davey Smith, and Burgess 2015). The MR‐PRESSO test, on the one hand, can test whether there is horizontal multiplicity and correct horizontal multiplicity by removing outliers; on the other hand, it can identify any significant change in causality before or after the removal of outliers (Marie et al. 2018). All analyses were carried out in R software (version 4.3.0) with the “TwoSample MR” and “MRPRESSO” packages (Hemani et al. 2018).

## 3 Results

### 3.1 The Causal Effect of Cognitive Impairment on Dysphagia

Based on the selection criteria for IVs, we identified 120 SNPs as IVs for MR analysis, and details of the SNPs are provided in Table 1. From Table 2, it can be seen that Cochran's *Q* test based on MR‐Egger and IVW found no evidence of heterogeneity in the IV effect (*Q* = 110.696, *p* = 0.694, IVW; *Q* = 107.996, *p* = 0.735, MR‐Egger), and thus the MR analysis adopted a fixed effects model.

**TABLE 1:** Details of the SNPs identified in cognitive impairment and dysphagia (forward).

SNP					Exposure (Cognitive Impairment)				Outcome (Dysphagia)			
RS ID	Chr	Position	EA	OA	Beta	Se	pval	EAF	Beta	Se	pval	EAF
rs1007934	14	73463479	A	G	0.016077	0.002806	1.00E−08	0.379905	0.0182	0.0246	0.4592	0.4828
rs10189857	2	60713235	A	G	0.018995	0.00275	4.91E−12	0.562889	0.0024	0.0247	0.9237	0.5195
rs10189912	2	1.44E+08	G	A	0.019337	0.002853	1.22E−11	0.353526	8.00E−04	0.0262	0.9754	0.3339
rs1054442	12	49389320	C	A	0.021463	0.002816	2.52E−14	0.380043	−0.0543	0.025	0.0295	0.4312
rs10779271	1	2.17E+08	A	G	0.016375	0.002925	2.17E−08	0.681229	−0.011	0.0263	0.6758	0.6731
rs10917152	1	22425642	T	C	0.024213	0.004049	2.23E−09	0.130936	2.00E−04	0.0442	0.9966	0.08632
rs10954779	8	31019597	C	T	0.016377	0.002762	3.04E−09	0.443548	−0.0445	0.0256	0.082621	0.3622
rs112780312	1	1.54E+08	G	A	0.018284	0.003099	3.66E−09	0.716492	−0.0069	0.0259	0.7913	0.6526
rs1144593	1	1.1E+08	G	A	0.019112	0.002987	1.57E−10	0.297398	0.003	0.0262	0.9082	0.3354
rs1145123	5	1.11E+08	T	C	0.020557	0.002772	1.20E−13	0.516307	0.0127	0.0253	0.617	0.4005
rs115064	7	24177191	T	C	0.016096	0.002814	1.07E−08	0.612319	0.0369	0.0265	0.1639	0.6844
rs11605348	11	47606483	G	A	0.016607	0.002896	9.73E−09	0.657869	0.0466	0.0266	0.079559	0.6408
rs11634187	15	40722781	T	G	0.022032	0.003857	1.12E−08	0.850447	−0.0085	0.0352	0.8079	0.8548
rs11646221	16	7666088	T	G	0.017735	0.002772	1.57E−10	0.55636	−0.0312	0.0251	0.2144	0.5917
rs11678106	2	82444107	T	C	0.016086	0.002745	4.62E−09	0.497074	0.0217	0.0247	0.3798	0.5132
rs11720523	3	71545170	A	C	0.018326	0.002773	3.89E−11	0.409655	−0.0105	0.0252	0.6765	0.3958
rs11793831	9	23362311	T	G	0.027834	0.002804	3.25E−23	0.40773	0.0024	0.0249	0.9246	0.4404
rs12035012	1	41750648	C	A	0.026992	0.003312	3.68E−16	0.782984	0.0477	0.0298	0.1097	0.781
rs1233578	6	28712247	G	A	0.023798	0.003903	1.08E−09	0.151324	0.0605	0.0525	0.2493	0.06064
rs12470949	2	23934816	C	T	0.017168	0.003021	1.32E−08	0.715753	0.0433	0.0264	0.1016	0.6834
rs12535854	7	1.05E+08	G	C	0.018226	0.002953	6.73E−10	0.663767	0.0151	0.0271	0.577801	0.7006
rs12646225	4	696848	T	C	0.025128	0.004215	2.51E−09	0.119864	−0.0022	0.0333	0.9477	0.1724
rs1280049	6	76536333	A	C	0.014993	0.002729	3.92E−08	0.475485	0.019	0.0248	0.443	0.4471
rs13024268	2	2.32E+08	G	A	0.016661	0.002879	7.15E−09	0.614167	0.0121	0.0252	0.6303	0.5838
rs13071190	3	1.37E+08	T	C	0.018095	0.002917	5.55E−10	0.667123	0.0279	0.0267	0.2969	0.6934
rs13212044	6	1.27E+08	G	T	0.018368	0.003242	1.46E−08	0.769433	0.0625	0.0317	0.04881	0.8126
rs13223152	7	69948241	A	G	0.017645	0.002784	2.34E−10	0.591423	0.0068	0.0247	0.7848	0.5298
rs13253386	8	14002020	G	T	0.020133	0.002748	2.37E−13	0.45967	0.0171	0.025	0.4953	0.4257
rs13276212	8	66440593	T	G	0.015071	0.002755	4.48E−08	0.482784	0.0032	0.0248	0.8968	0.4784
rs1362739	7	1.33E+08	A	C	0.020945	0.002734	1.83E−14	0.468787	0.0184	0.0252	0.4647	0.4079
rs1589652	3	35538452	A	G	0.017094	0.002759	5.82E−10	0.443301	−0.0028	0.0249	0.9119	0.4377
rs166820	5	89353210	A	G	0.024334	0.003599	1.37E−11	0.173576	0.0382	0.0322	0.2358	0.1788
rs17002025	19	12530177	A	G	0.025598	0.004261	1.89E−09	0.12239	0.0573	0.0457	0.2107	0.08002
rs17106817	14	69716957	T	C	0.016911	0.003025	2.26E−08	0.706668	0.0315	0.0282	0.2641	0.7433
rs1727307	12	1.24E+08	A	G	0.017817	0.003007	3.10E−09	0.288605	−0.0271	0.0279	0.3306	0.2679
rs1812587	5	62991802	G	T	0.017339	0.002767	3.68E−10	0.517493	4.00E−05	0.025	0.9816	0.449
rs1831539	1	59560337	C	T	0.017203	0.002762	4.72E−10	0.457191	0.0055	0.0248	0.8248	0.4693
rs1840847	5	13100175	A	G	0.016342	0.002883	1.44E−08	0.350354	−0.0177	0.0248	0.4751	0.4609
rs1891273	10	93442379	T	C	0.015414	0.002786	3.17E−08	0.48879	0.0251	0.0256	0.3258	0.6245
rs1906252	6	98550289	A	C	0.031662	0.002741	7.48E−31	0.465953	0.0128	0.0247	0.6061	0.4735
rs1962047	12	58292707	G	A	0.019533	0.002863	8.89E−12	0.63782	−0.0194	0.0251	0.4408	0.5897
rs1972860	4	94579640	G	A	0.017556	0.00293	2.09E−09	0.679165	−0.0028	0.0265	0.9163	0.6841
rs2008514	16	28825605	G	A	0.028678	0.002799	1.25E−24	0.61526	−0.0194	0.025	0.4387	0.5863
rs2071407	14	1.04E+08	C	T	0.021974	0.002859	1.52E−14	0.637542	−0.0156	0.0255	0.541	0.6247
rs2072490	19	18257750	T	C	0.016996	0.002745	5.93E−10	0.509332	0.0495	0.0247	0.04526	0.504
rs2111490	8	1.05E+08	A	G	0.015491	0.002753	1.83E−08	0.464706	0.0134	0.0249	0.5904	0.4317
rs2239647	14	33292743	C	A	0.020537	0.002766	1.14E−13	0.552217	0.0162	0.025	0.517101	0.5706
rs2268894	2	1.63E+08	T	C	0.020785	0.002749	3.98E−14	0.546889	−0.004	0.0253	0.8746	0.6032
rs2285640	17	34951204	A	G	0.017514	0.002765	2.38E−10	0.544102	0.0343	0.0249	0.1679	0.5602
rs2309812	2	1.01E+08	T	C	0.022835	0.002845	9.95E−16	0.358192	−0.008	0.0253	0.752101	0.4014
rs2373353	11	79162622	G	A	0.016323	0.002887	1.56E−08	0.362827	−0.0062	0.0252	0.8047	0.4055
rs2393967	10	65133156	C	A	0.018709	0.002963	2.70E−10	0.308516	0.0206	0.0269	0.4433	0.2994
rs2450333	5	1.77E+08	G	A	0.018829	0.002799	1.73E−11	0.50502	−0.025	0.0249	0.3141	0.5369
rs2457192	16	12197441	C	A	0.019751	0.003131	2.84E−10	0.276732	4.00E−04	0.0278	0.9873	0.2778
rs2478286	13	1.07E+08	G	C	0.025785	0.003127	1.64E−16	0.254296	0.0039	0.0286	0.8911	0.2487
rs2508713	11	95547927	A	T	0.016531	0.002841	5.92E−09	0.361565	0.0106	0.0284	0.7098	0.2521
rs2647995	16	51577196	C	T	0.019749	0.003044	8.68E−11	0.29458	−0.0447	0.0271	0.09899	0.3013
rs2678210	1	2.02E+08	T	C	0.018786	0.003046	6.97E−10	0.714044	0.0171	0.0272	0.5302	0.7086
rs2726491	4	1.06E+08	G	A	0.02828	0.002857	4.17E−23	0.649584	−0.041	0.0261	0.1159	0.6605
rs2836921	21	40516070	A	G	0.020346	0.002963	6.54E−12	0.312288	0.0795	0.03	0.008012	0.2186
rs28620532	9	98216876	G	A	0.016354	0.002889	1.51E−08	0.35636	−0.0138	0.0256	0.5903	0.3672
rs287879	6	1.57E+08	G	A	0.018867	0.003075	8.47E−10	0.269218	0.043	0.031	0.1648	0.1983
rs2920940	8	93180965	C	T	0.024742	0.003252	2.76E−14	0.76931	2.00E−05	0.03	0.9919	0.7834
rs2955280	2	44116836	C	T	0.014915	0.002734	4.90E−08	0.472036	0.0032	0.0248	0.8966	0.5053
rs3128341	1	72749848	C	T	0.031727	0.003417	1.63E−20	0.801925	0.0024	0.0301	0.9358	0.7853
rs31768	5	1.65E+08	A	T	0.018177	0.003054	2.65E−09	0.286649	−0.0248	0.0262	0.343	0.3349
rs329672	11	1.34E+08	T	C	0.01743	0.002853	1.00E−09	0.627764	−0.0232	0.0256	0.3647	0.6339
rs34316	5	88015545	A	C	0.021049	0.002767	2.82E−14	0.434694	−0.0308	0.025	0.2183	0.4264
rs34320898	1	1.72E+08	C	G	0.022869	0.003844	2.70E−09	0.183646	0.007	0.0319	0.8272	0.1829
rs34811474	4	25408838	A	G	0.028996	0.003594	7.15E−16	0.19789	−0.0242	0.0295	0.4117	0.2308
rs35608616	10	1.25E+08	G	A	0.018089	0.002937	7.33E−10	0.66948	0.0134	0.027	0.6188	0.6983
rs35731967	2	2E+08	T	C	0.021838	0.003658	2.38E−09	0.819864	−0.0338	0.0329	0.3048	0.8285
rs3740422	10	1.04E+08	G	C	0.024102	0.002912	1.25E−16	0.673714	−0.024	0.0259	0.3539	0.6517
rs3843954	13	58548511	G	C	0.020758	0.003343	5.31E−10	0.725839	−0.0489	0.0281	0.081579	0.7379
rs3860537	3	1.08E+08	T	C	0.018854	0.003402	2.99E−08	0.209994	0.0168	0.0304	0.5797	0.2101
rs3896224	10	1.06E+08	G	A	0.015315	0.002772	3.29E−08	0.445642	0.0269	0.0247	0.2765	0.493
rs4463213	5	1.4E+08	A	G	0.019065	0.002732	3.00E−12	0.520681	−0.0063	0.0246	0.7968	0.5053
rs4725065	7	8109522	G	A	0.016532	0.002736	1.52E−09	0.481968	0.0079	0.0253	0.754699	0.3907
rs4793161	17	42919009	G	A	0.017719	0.00325	4.97E−08	0.767539	0.0443	0.0287	0.1231	0.7567
rs4852252	2	71539301	C	T	0.020787	0.002747	3.84E−14	0.55	−0.0323	0.0247	0.1911	0.5323
rs4976976	8	1.43E+08	A	G	0.017317	0.002777	4.53E−10	0.419172	−0.0091	0.0248	0.713199	0.4384
rs55754731	12	15532891	T	C	0.021369	0.003675	6.06E−09	0.831337	−0.0137	0.0348	0.6951	0.853
rs56150095	7	71759069	C	A	0.021968	0.002747	1.28E−15	0.468124	−0.0011	0.0247	0.9643	0.5008
rs566237	6	11543342	G	A	0.018716	0.002935	1.82E−10	0.316739	0.0173	0.0281	0.537901	0.262
rs5750830	22	39840828	A	C	0.022891	0.003127	2.46E−13	0.741484	0.0104	0.0281	0.709999	0.7399
rs6019535	20	47541517	A	G	0.025105	0.002976	3.28E−17	0.305297	−0.0451	0.028	0.1067	0.2624
rs60262711	2	1.18E+08	T	C	0.015949	0.002825	1.65E−08	0.384878	0.023	0.0251	0.3584	0.4111
rs62181012	2	1.81E+08	T	C	0.021146	0.003511	1.73E−09	0.807961	0.068	0.0305	0.02577	0.7931
rs6508220	18	50831176	G	A	0.022748	0.002737	9.56E−17	0.496782	−0.0045	0.0249	0.8572	0.4375
rs6535809	4	1.53E+08	A	G	0.019647	0.002734	6.65E−13	0.513351	−0.0134	0.0247	0.5874	0.534
rs6539284	12	79592680	C	T	0.019481	0.002827	5.56E−12	0.387003	0.0154	0.025	0.5383	0.4183
rs6550835	3	24062661	G	A	0.024809	0.002929	2.44E−17	0.673653	0.001	0.0256	0.9693	0.6335
rs6668048	1	96199657	C	T	0.021458	0.002734	4.24E−15	0.528149	−0.0246	0.0249	0.322	0.5728
rs66954617	17	57004787	G	A	0.020882	0.002834	1.72E−13	0.625562	0.0213	0.0259	0.411	0.6521
rs67482514	4	65785448	G	C	0.017859	0.003229	3.21E−08	0.24384	4.00E−06	0.0281	0.9906	0.2606
rs6770622	3	85171415	G	A	0.044961	0.006864	5.76E−11	0.958254	−0.0039	0.077	0.9592	0.97353
rs6819372	4	67970101	G	A	0.019796	0.002729	4.02E−13	0.507391	−0.0136	0.0249	0.5858	0.4487
rs6903716	6	21956404	A	G	0.017758	0.002975	2.39E−09	0.700015	−0.0195	0.0262	0.4578	0.6623
rs7069887	10	29569272	A	C	0.022532	0.003898	7.44E−09	0.852171	0.0184	0.0377	0.6256	0.8779
rs7116046	11	1.06E+08	T	C	0.015707	0.002842	3.27E−08	0.371189	0.0088	0.0249	0.7248	0.422
rs7172979	15	51817198	T	G	0.060634	0.009084	2.47E−11	0.023837	0.0491	0.1385	0.7227	0.007787
rs7248006	19	31929180	C	T	0.019175	0.00282	1.05E−11	0.612134	−0.0139	0.0248	0.5758	0.5228
rs72739469	15	65738080	C	T	0.034357	0.005648	1.18E−09	0.068925	0.0667	0.0405	0.099529	0.1047
rs73068339	19	59090263	C	G	0.018858	0.003046	5.96E−10	0.282707	−0.01	0.0265	0.7067	0.3228
rs7312919	12	92960905	C	G	0.018146	0.002915	4.83E−10	0.662565	−0.0444	0.0248	0.07383	0.5568
rs7357604	8	1.43E+08	A	G	0.015704	0.00282	2.56E−08	0.621728	−0.0241	0.0254	0.3428	0.6227
rs75973558	5	26880925	A	G	0.025636	0.004465	9.42E−09	0.884555	−0.0436	0.0466	0.3494	0.92178
rs78084033	20	34219990	C	A	0.022875	0.004049	1.62E−08	0.13437	−0.0078	0.0375	0.836	0.124
rs7941785	11	63861317	A	G	0.015512	0.002841	4.75E−08	0.36911	0.0043	0.0259	0.8678	0.3473
rs799444	7	44769190	T	C	0.018415	0.002759	2.48E−11	0.450816	−0.0282	0.0253	0.265	0.6029
rs8006700	14	27162904	T	A	0.018227	0.00293	4.96E−10	0.320588	0.0084	0.0297	0.7769	0.224
rs8025964	15	82521770	A	G	0.017031	0.002749	5.78E−10	0.465383	0.0354	0.0247	0.1514	0.4749
rs8051038	16	71870700	A	G	0.018924	0.003146	1.78E−09	0.749415	0.0129	0.0294	0.659601	0.7702
rs8054299	16	53498655	G	C	0.023006	0.002927	3.84E−15	0.317524	0.0112	0.0281	0.6897	0.2646
rs889169	19	47548678	A	G	0.016071	0.002892	2.75E−08	0.600693	0.0167	0.0261	0.5212	0.6618
rs913264	9	1.32E+08	T	C	0.019725	0.003026	7.09E−11	0.286634	−0.0165	0.0287	0.5652	0.2506
rs9384679	6	1.09E+08	C	T	0.026724	0.002783	7.94E−22	0.601216	−0.048	0.025	0.055151	0.5786
rs9503599	6	3451048	C	T	0.017111	0.002785	8.05E−10	0.437234	0.0263	0.025	0.2935	0.4156
rs967569	2	41616346	C	T	0.017976	0.002927	8.21E−10	0.325993	0.0438	0.0288	0.1284	0.2416
rs9888986	16	68297228	G	A	0.023502	0.004262	3.52E−08	0.883708	0.0414	0.036	0.2498	0.8631

**TABLE 2:** Sensitivity analysis of MR Analysis.

	Heterogeneity tests		Directional horizontal pleiotropy						
	MR‐Egger		IVW		MR‐Egger			MR‐PRESSO global pleiotropy test	
Outcome	*Q*	pval	*Q*	pval	Intercept	SE	pval	RSSobs	pval
Dysphagia	107.996	0.735	110.696	0.694	0.0211	0.0129	0.1030	0.073	0.54

Abbreviations: IVW, inverse variance weighting, MR, Mendelian randomization, pval, *p* value, *Q*, Cochran's *Q* test, SE, standard error.

**FIGURE 2:**
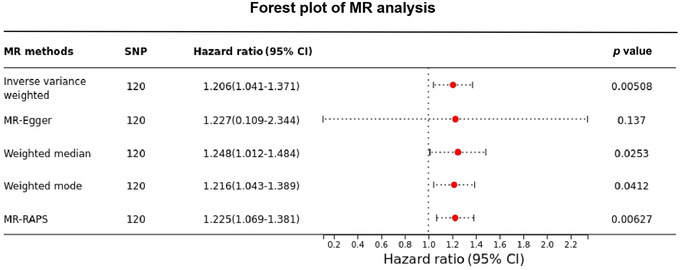
Forest plot showing detailed estimates of the causal effect of the association between cognitive impairment and dysphagia in different models.

Next, we assessed the causal effect of cognitive impairment on dysphagia by five MR analyses, as presented in Figure 2. IVW results indicated that cognitive impairment was associated with an increased risk of dysphagia (OR: 1.206, 95% CI: [1.041, 1.371], *p* = 0.00508), and there was a causal link between the two. Meanwhile, we validated this finding by weighted median (OR: 1.248, 95% CI: [1.012, 1.484], *p* = 0.0253), weighted mode (OR: 1.216, 95% CI: [1.043, 1.389], *p* = 0.0412), and MR‐RAPS (OR: 1.225, 95% CI: [1.069, 1.381], *p* = 0.00627).

### 3.2 Sensitivity Analysis

**FIGURE 3:**
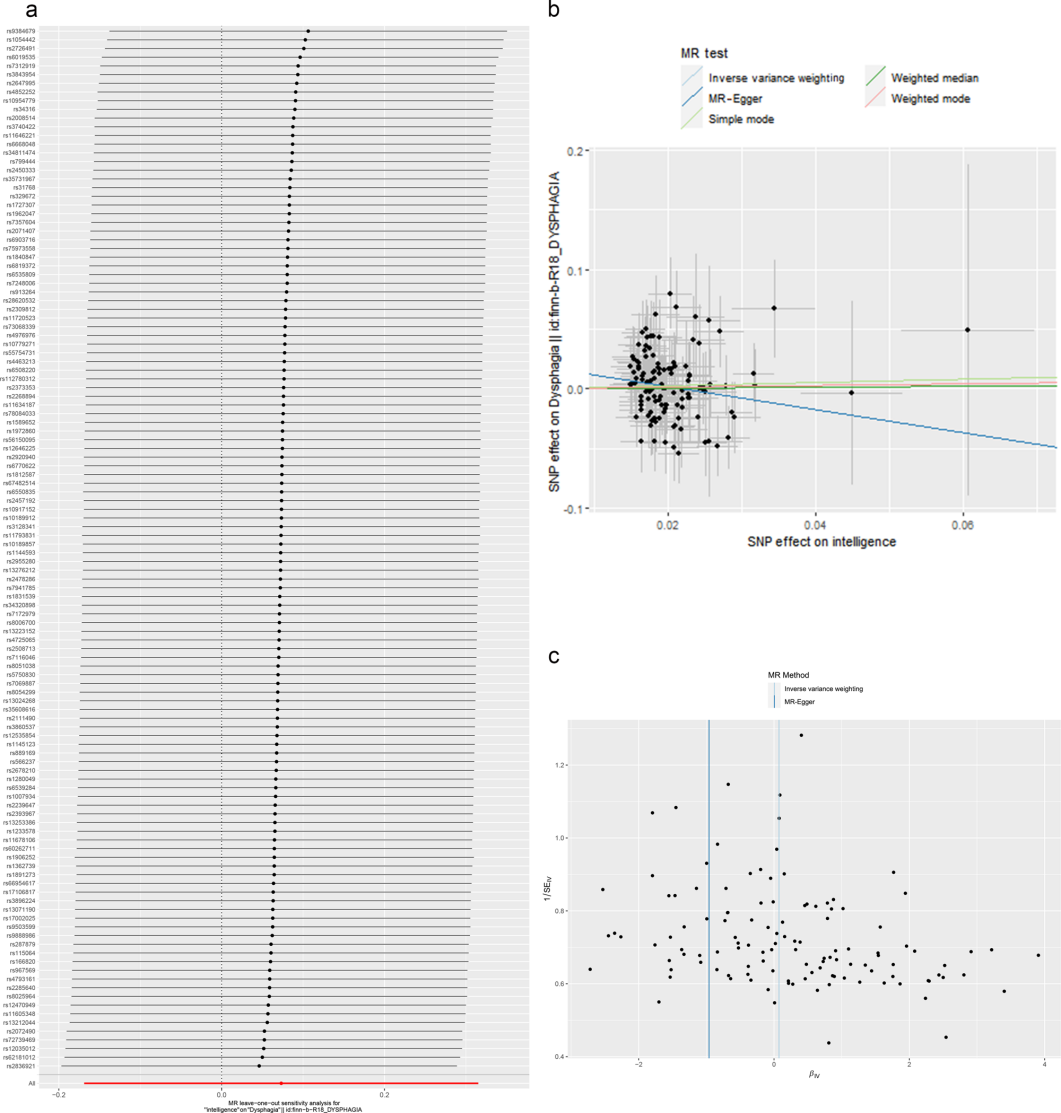
(a) Detail graph of the leave‐one‐out sensitivity tests. The residual IVs were eliminated one by one and the MR results of the rest IVs were computed. (b) Causality scatter plot. The slope of each line corresponds to the MR effect evaluated in the different models. (c) Funnel plot to detect whether the observed correlations are significantly heterogeneous.

We then conducted multiple sensitivity analyses to verify the robustness of the above causal effects. The *F*‐statistics of all 120 selected IVs were above 10, and the *R* ^2^ was less than 0.001, indicating that the selected instruments were all strong IVs. In addition, the *p* values for both the MR‐Egger intercept (*p* = 0.103) and the MR‐PRESSO global test (*p* = 0.540) were over 0.05, suggesting no significant horizontal pleiotropy between cognitive impairment and risk of dysphagia (Table 2). The leave‐one‐out (LOO) analysis revealed that the cause–effect relationship was not driven by a single IV, which confirmed the robustness of the findings (Figure 3a). Besides, scatter plot analysis not only demonstrated that there was no causal effect between cognitive impairment and dysphagia but also suggested the absence of heterogeneity across the chosen SNPs (Figure 3b). Similarly, the funnel plot also confirmed that there was no significant heterogeneity between the SNPs selected for the two disorders, further supporting the robustness of the MR results (Figure 3c).

## 4 Discussion

We first systematically investigated the potential causal relationship between cognitive impairment and dysphagia using MR methods. In this study, we estimated the causal effect between cognitive impairment and dysphagia based on a large database of GWAS meta‐analyses and multiple complementary MR analysis methods. Then, the results of multiple MR analyses showed that cognitive impairment had a significant causal effect on the risk of dysphagia. Sensitivity tests such as multiplicity and heterogeneity were also performed to ensure the accuracy and robustness of the MR results. In summary, the results of this study suggest that the risk of dysphagia is directly related to cognitive impairment and that early preventive intervention for dysphagia should be provided for people with cognitive impairment.

In this research, we employed several methods to test the hypotheses of MR to ensure the reliability of the results. First, to satisfy the assumption of association, SNPs that were strongly correlated with exposure factors were selected as IVs in this study, conditional on a *p* < 5 × 10^−8^, and weakly correlated variables were removed by the screening criterion of *F*‐test value > 10. Secondly, we removed the linkage disequilibrium with the criteria of *r* ^2^ < 0.001 and kb > 1000 to ensure that the two SNPs were randomly assigned and independent of each other. Thus, the independence assumption was met. Third, the MR‐Egger intercept test and MR‐PRESSO were conducted, and the *p* value was greater than 0.05, indicating that the IVs were not pleiotropic. It suggests that SNPs contribute to the outcome only through exposure factors, which means that the exclusion hypothesis is satisfied (Bowden, Davey Smith, and Burgess 2015; Marie et al. 2018).

Previously, meta‐analyses of many observational studies have shown a significant association between cognitive impairment and the development of dysphagia (Dehaghani, Doosti, and Zare 2021). Compared with patients without cognitive impairment, patients with cognitive impairment had worse masticatory and swallowing functions (Chen et al. 2021; De Stefano et al. 2020). Several studies have consistently shown a significant positive correlation between the severity of cognitive decline and dysphagia, with cortical involvement and severity worsening dysphagia in patients with cognitive impairment (Al Rjoob and Al Rjoob 2022; Seçil et al. 2016). For instance, the prevalence of dysphagia was 16.7% in subjects with mild to moderate Alzheimer's disease and 91.8% in subjects with severe AD (Simões, Oliva Filho, and Hebling 2020). These are consistent with our findings. In the present study, we used five complementary MR methods to investigate that there is a direct causal effect between cognitive impairment and dysphagia (OR: 1.206, IVW, as shown in Figure 2), which means that cognitive impairment can lead to the development of dysphagia. Similarly, it has been found that swallowing‐related brain regions are more susceptible to damage in Alzheimer's patients, and that worsening cognitive impairment may exacerbate dysphagia (Chouinard 2000; Ebrahimian Dehaghani et al. 2019; Malandraki et al. 2009; Martin et al. 2007). In addition, evidence also suggests that cognitive dysfunction may be an independent predictor of dysphagia (Yokota et al. 2016). In summary, these findings suggest that cognitive impairment may contribute to the development of dysphagia.

Similar biological mechanisms may help explain the causal relationship between cognitive impairment and dysphagia. First, both swallowing behavior and cognitive function are importantly regulated by the frontal lobes and other cortical areas (Friedman and Robbins 2022; Qiao et al. 2022). In Alzheimer's disease, both swallowing and cognition involve multiple neuroanatomical systems, with areas of involvement including supramedullary structures, especially the superior temporal gyrus, insula, cingulate gyrus, and parieto–occipital association areas (Rösler et al. 2015). Second, several studies have noted that cognitive domains such as visual attention and executive function have an impact on certain phases of swallowing, like the oral phase (Jo, Hwang, and Pyun 2017). For example, Suh, Kim, and Na (2009) reported that the reduced perception of food in the mouth may result in delayed oral delivery. This results in patients taking longer to transfer food to the pharynx, leading to an increase in the oral delivery time. In individuals with Alzheimer's disease, deficits in swallowing sensation lead to reduced taste sensation and impaired sense of smell (Suh, Kim, and Na 2009). Third, there is evidence for cortico–cortical connections between related motor, sensory, and higher convergent areas that play a role in behavioral theories of perception of hand movements and oral movements (Pulvermüller et al. 2014). A study of patients with Parkinson's disease and dysphagia noted that dysphagia may stem from underlying executive dysfunction in cognitive domains, which may partially explain the association between cognition and swallowing (Roberts et al. 2017). Finally, studies of motor imagery (a cognitive strategy) during swallowing have provided additional evidence for a significant relationship between swallowing and cognition. Swallowing function has a motor component controlled by the motor cortex and premotor cortex, two areas that are critical during motor imagery and can influence higher‐level cognitive processing (Cheng et al. 2022; Madan and Singhal 2012; H. Yang et al. 2016).

However, some studies have yielded conflicting results, suggesting that there is no link between cognitive impairment and dysphagia (Holland et al. 2011; Maeda et al. 2018; Michel et al. 2018). For example, in an observational study on older adults, no significant correlation was found between cognitive functions such as memory, recall, and mental performance and dysphagia (Holland et al. 2011). In addition, one study noted no significant difference in the likelihood of dysphagia in women with amnesic or non‐amnesic cognitive impairment compared to women without cognitive impairment (E. Yang et al. 2014). These are inconsistent with the results of the present study, possibly because observational studies are often confounded by small sample sizes, potential confounders, or reverse causality. MR studies, which rely on genetic variation, use genetic variation that is strongly correlated with exposure as an IV to infer causal links between exposure and outcome. Since gametes are formed according to the Mendelian law of inheritance, in which parental alleles are randomly assigned to offspring, genetic variants are not affected by traditional confounders such as environmental exposures, socioeconomic status, and behaviors. Furthermore, genetic variants are inherited from parents and remain unchanged from birth, and their associations with outcomes are chronologically plausible. Thus, MR is able to overcome the confounding and reverse causation problems that exist in traditional observational epidemiologic studies (Davey Smith and Hemani 2014). In the present MR study, we innovatively included a total of 470,363 Europeans based on the authoritative GWAS database and used five different and complementary MR analysis methods to demonstrate a significant causal effect between cognitive impairment and dysphagia while also eliminating the interference of confounders. This finding has important implications for the clinical management of both disorders.

### 4.1 Strengths and Limitations of This Study

In addition to this, there are a couple of strengths in our study. First, the present study provides the first MR analysis research to explore the causality of cognitive impairment and dysphagia, which provides a basis for further exploration of the genetic causality between cognitive impairment and dysphagia. Second, the summary statistics of cognitive impairment and dysphagia in this study were acquired from the GWAS database, with a sufficiently large sample size, and analyzed by a two‐sample MR method, which allows for greater reduction of the effects of confounders and reverse causation, thus yielding reliable causal associations. Third, five different complementary models were applied in this study to replicate the identification of causality between cognitive impairment and dysphagia, all of which led to the same conclusion. Fourth, sensitivity testing using various methods such as heterogeneity analysis, sample overlap effect estimation, and horizontal multivariate validity in this study all suggested that causality resulting from MR analyses was not interfered with by confounding factors, which confirmed the soundness and precision of the MR results.

Inevitably, however, there are some limitations to this study. First, given the potential confounding by heterogeneous populations, our MR analyses included only those of European origin, which may limit our confidence in generalizing our results to other ethnicities. Second, there may be overlapping participants in both exposure and outcome research, the degree of which is difficult to estimate. Fortunately, however, this study used powerful tools, such as *F*‐statistics greater than 10, to minimize the possible bias caused by sample duplication (Pierce and Burgess 2013). Finally, what is noteworthy is that due to the lack of data on the subtypes of patients with dysphagia and cognitive impairment, this study could not further stratify the patients, and therefore, the findings need to be cautiously interpreted, particularly in the context of clinic practice and decision‐making.

In the future, for a better understanding of the mutual interaction between dysphagia and cognitive impairment, first of all, larger prospective studies based on different ethnicities are recommended to figure out whether there is causality between cognitive impairment and dysphagia. Second, further exploration of the interactions between different subtypes of cognitive impairment and different subtypes of dysphagia would be beneficial to enhance our in‐depth knowledge of the association between the two. Third, future research on the mechanisms between cognitive impairment and dysphagia could focus on the multiple perspectives, such as cognitive function, neurotransmitters, and functional reorganization of brain networks, to explore how cognitive impairment affects the initiation, execution, and control of swallowing function through methods such as videofluoroscopic swallowing study and neurostimulation techniques.

As suggested by the associated OR (OR: 1.206, IVW) in this study, cognitive impairment is a detrimental factor for dysphagia. Based on this, when developing a treatment plan for patients with cognitive impairment, besides the dual assessment of cognitive and swallowing functions, an individualized rehabilitation and care plan should be established for patients who may suffer from dysphagia, such as closely monitoring the nutritional and hydration status of the patient, strengthening swallowing training, and reducing the risk of aspiration. Specifically, to improve the feeding ability and quality of life of patients with cognitive impairment and to decrease the risk of dysphagia, we recommend the following measures: First, provide a quiet, distraction‐free dining environment for patients with cognitive impairment and remove extraneous stimuli from the environment to minimize distraction. Second, caregivers should offer good assistance such as verbal prompts, encouragement, and positive feedback during the meal. Third, appropriate utensils and foods help stimulate the patient's senses and increase pharyngeal sensitivity to facilitate food intake and swallowing (Ney et al. 2009).

## 5 Conclusion

Our findings suggest that cognitive impairment may increase the risk of dysphagia from a genetic inheritance perspective. This not only helps us to better understand the genetic link between these two disorders but also provides new ideas for clinicians to diagnose and treat cognitive impairment and dysphagia. When treating patients with cognitive impairment, clinicians should pay attention to the symptoms of dysphagia, which can help to choose better treatments and make early interventions. However, further clinical studies are needed to replicate these findings, and further research is necessary to explore medications or other therapies for treating comorbidities of cognitive impairment and dysphagia and to elucidate the underlying mechanisms of the comorbidities.

## Author Contributions

**Yueqin Tian**: conceptualization, investigation, methodology, formal analysis, project administration, writing–original draft, writing–review and editing. **Jiahui Hu**: data curation, project administration, formal analysis, writing–original draft. **Qianqian Wang**: methodology, formal analysis, writing–review and editing. **Jia Qiao**: formal analysis, writing–review and editing. **Hongmei Wen**: formal analysis, writing–review and editing. **Qiuping Ye**: conceptualization, project administration, supervision, funding acquisition, writing–review and editing. **Zulin Dou**: writing–review and editing, funding acquisition, conceptualization, project administration, supervision.

## Ethics Statement

This study was a secondary analysis of publicly available GWAS summary data and did not use individual‐level data, so no new ethics committee approval was required.

## Consent

The authors have nothing to report.

## Conflicts of Interest

The authors declare that they have no competing interests.

### Peer Review

The peer review history for this article is available at https://publons.com/publon/10.1002/brb3.70295.

## Data Availability

All the datasets analyzed during the current study are publicly available in the GWAS summary data repository. Raw datasets for dysphagia can be downloaded at FinnGen base (GWAS ID: finn‐b‐R18_DYSPHAGIA, https://gwas.mrcieu.ac.uk/datasets/finn‐b‐R18_DYSPHAGIA/). Genetic data on cognitive impairment were obtained from a GWAS meta‐analysis of 269,867 individuals of European ethnicity, which identified 205 genomic loci strongly associated with cognition (Savage et al. 2018). More details are available from the corresponding author upon reasonable request.

## References

[brb370295-bib-0001] Adkins, C. , W. Takakura , B. M. R. Spiegel , et al. 2020. “Prevalence and Characteristics of Dysphagia Based on a Population‐Based Survey.” Clinical Gastroenterology and Hepatology 18, no. 9: 1970–1979.e2. 10.1016/j.cgh.2019.10.029. 31669055 PMC7180111

[brb370295-bib-0002] Airoldi, M. , M. Garzaro , L. Raimondo , et al. 2011. “Functional and Psychological Evaluation After Flap Reconstruction Plus Radiotherapy in Oral Cancer.” Head & Neck 33, no. 4: 458–468. 10.1002/hed.21471. 20652979

[brb370295-bib-0003] Al Rjoob, M. , and K. Al Rjoob . 2022. “The Correlation Between Cognitive Function and Dysphagia in Stroke Patients.” La Tunisie Medicale 100, no. 4: 342–345. 36155906 PMC9579867

[brb370295-bib-0004] Bang, J. , S. Spina , and B. L. Miller . 2015. “Frontotemporal Dementia.” Lancet 386, no. 10004: 1672–1682. 10.1016/S0140-6736(15)00461-4. 26595641 PMC5970949

[brb370295-bib-0005] Berron, D. , K. Neumann , A. Maass , et al. 2018. “Age‐Related Functional Changes in Domain‐Specific Medial Temporal Lobe Pathways.” Neurobiology of Aging 65: 86–97. 10.1016/j.neurobiolaging.2017.12.030. 29454154

[brb370295-bib-0006] Bowden, J. , G. Davey Smith , and S. Burgess . 2015. “Mendelian Randomization With Invalid Instruments: Effect Estimation and Bias Detection Through Egger Regression.” International Journal of Epidemiology 44, no. 2: 512–525. 10.1093/ije/dyv080. 26050253 PMC4469799

[brb370295-bib-0007] Bowden, J. , G. Davey Smith , P. C. Haycock , and S. Burgess . 2016. “Consistent Estimation in Mendelian Randomization With Some Invalid Instruments Using a Weighted Median Estimator.” Genetic Epidemiology 40, no. 4: 304–314. 10.1002/gepi.21965. 27061298 PMC4849733

[brb370295-bib-0008] Bowden, J. , and M. V. Holmes . 2019. “Meta‐Analysis and Mendelian Randomization: A Review.” Research Synthesis Methods 10, no. 4: 486–496. 10.1002/jrsm.1346. 30861319 PMC6973275

[brb370295-bib-0009] Brion, M.‐J. A. , K. Shakhbazov , and P. M. Visscher . 2013. “Calculating Statistical Power in Mendelian Randomization Studies.” International Journal of Epidemiology 42, no. 5: 1497–1501. 10.1093/ije/dyt179. 24159078 PMC3807619

[brb370295-bib-0010] Burgess, S. 2014. “Sample Size and Power Calculations in Mendelian Randomization With a Single Instrumental Variable and a Binary Outcome.” International Journal of Epidemiology 43, no. 3: 922–929. 10.1093/ije/dyu005. 24608958 PMC4052137

[brb370295-bib-0011] Burgess, S. , A. Butterworth , and S. G. Thompson . 2013. “Mendelian Randomization Analysis With Multiple Genetic Variants Using Summarized Data.” Genetic Epidemiology 37, no. 7: 658–665. 10.1002/gepi.21758. 24114802 PMC4377079

[brb370295-bib-0012] Burgess, S. , D. S. Small , and S. G. Thompson . 2017. “A Review of Instrumental Variable Estimators for Mendelian Randomization.” Statistical Methods in Medical Research 26, no. 5: 2333–2355. 10.1177/0962280215597579. 26282889 PMC5642006

[brb370295-bib-0013] Burgess, S. , S. G. Thompson , and CRP CHD Genetics Collaboration . 2011. “Avoiding Bias From Weak Instruments in Mendelian Randomization Studies.” International Journal of Epidemiology 40, no. 3: 755–764. 10.1093/ije/dyr036. 21414999

[brb370295-bib-0014] Cao, Q. , C. C. Tan , W. Xu, et al. 2020. “The Prevalence of Dementia: A Systematic Review and Meta‐Analysis.” Journal of Alzheimer's Disease 73, no. 3: 1157–1166. 10.3233/JAD-191092. 31884487

[brb370295-bib-0015] Chen, L. , L. Gu , X. Li , W. Chen , and L. Zhang . 2021. “Oral Health Matters in Cognitive Impaired Aged Residents in Geriatric Care Facilities: A Cross‐Sectional Survey.” Nursing Open 8, no. 2: 792–798. 10.1002/nop2.683. 33570297 PMC7877127

[brb370295-bib-0016] Cheng, I. , K. Takahashi , A. Miller , and S. Hamdy . 2022. “Cerebral Control of Swallowing: An Update on Neurobehavioral Evidence.” Journal of the Neurological Sciences 442: 120434. 10.1016/j.jns.2022.120434. 36170765

[brb370295-bib-0017] Chouinard, J. 2000. “Dysphagia in Alzheimer Disease: A Review.” Journal of Nutrition, Health & Aging 4, no. 4: 214–217. 11115803

[brb370295-bib-0018] Cichero, J. A. Y. , P. Lam , C. M. Steele , et al. 2017. “Development of International Terminology and Definitions for Texture‐Modified Foods and Thickened Fluids Used in Dysphagia Management: The IDDSI Framework.” Dysphagia 32, no. 2: 293–314. 10.1007/s00455-016-9758-y. 27913916 PMC5380696

[brb370295-bib-0019] Cichero, J. A. Y. , C. Steele , J. Duivestein, et al. 2013. “The Need for International Terminology and Definitions for Texture‐Modified Foods and Thickened Liquids Used in Dysphagia Management: Foundations of a Global Initiative.” Current Physical Medicine and Rehabilitation Reports 1, no. 4: 280–291. 10.1007/s40141-013-0024-z. 24392282 PMC3873065

[brb370295-bib-0020] Collin, S. H. P. , B. Milivojevic , and C. F. Doeller . 2015. “Memory Hierarchies Map Onto the Hippocampal Long Axis in Humans.” Nature Neuroscience 18, no. 11: 1562–1564. 10.1038/nn.4138. 26479587 PMC4665212

[brb370295-bib-0021] Coppin, G. 2016. “The Anterior Medial Temporal Lobes: Their Role in Food Intake and Body Weight Regulation.” Physiology & Behavior 167: 60–70. 10.1016/j.physbeh.2016.08.028. 27591841

[brb370295-bib-0022] Cristofori, I. , S. Cohen‐Zimerman , and J. Grafman . 2019. “Executive Functions.” Handbook of Clinical Neurology 163: 197–219. 10.1016/B978-0-12-804281-6.00011-2. 31590731

[brb370295-bib-0023] Davey Smith, G. , and G. Hemani . 2014. “Mendelian Randomization: Genetic Anchors for Causal Inference in Epidemiological Studies.” Human Molecular Genetics 23, no. R1: R89–R98. 10.1093/hmg/ddu328. 25064373 PMC4170722

[brb370295-bib-0024] Davies, N. M. , M. V. Holmes , and G. Davey Smith . 2018. “Reading Mendelian Randomisation Studies: A Guide, Glossary, and Checklist for Clinicians.” BMJ 362: k601. 10.1136/bmj.k601. 30002074 PMC6041728

[brb370295-bib-0025] Defenderfer, J. , A. Kerr‐German , M. Hedrick , and A. T. Buss . 2017. “Investigating the Role of Temporal Lobe Activation in Speech Perception Accuracy With Normal Hearing Adults: An Event‐Related fNIRS Study.” Neuropsychologia 106: 31–41. 10.1016/j.neuropsychologia.2017.09.004. 28888891

[brb370295-bib-0026] Dehaghani, S. E. , A. Doosti , and M. Zare . 2021. “Association Between Swallowing Disorders and Cognitive Disorders in Adults: A Systematic Review and Meta‐Analysis.” Psychogeriatrics 21, no. 4: 668–674. 10.1111/psyg.12704. 33934446

[brb370295-bib-0027] Dell'Aquila, G. , N. J. Peladic , V. Nunziata , et al. 2022. “Prevalence and Management of Dysphagia in Nursing Home Residents in Europe and Israel: The SHELTER Project.” BMC Geriatrics 22, no. 1: 719. 10.1186/s12877-022-03402-y. 36042405 PMC9429699

[brb370295-bib-0028] De Stefano, A. , P. Di Giovanni , G. Kulamarva, et al. 2020. “Oropharyngeal Dysphagia in Elderly Population Suffering From Mild Cognitive Impairment and Mild Dementia: Understanding the Link.” American Journal of Otolaryngology 41, no. 4: 102501. 10.1016/j.amjoto.2020.102501. 32409161

[brb370295-bib-0029] Ebrahimian Dehaghani, S. , F. Yadegari , A. Asgari , and Z. Bagheri . 2019. “The Mediator Effect of Cognition on the Relationship Between Brain Lesion Location and Dysphagia in Patients With Stroke: Applying a Structural Equation Model.” Journal of Oral Rehabilitation 46, no. 1: 33–39. 10.1111/joor.12722. 30252946

[brb370295-bib-0030] Emdin, C. A. , A. V. Khera , and S. Kathiresan . 2017. “Mendelian Randomization.” JAMA 318, no. 19: 1925–1926. 10.1001/jama.2017.17219. 29164242

[brb370295-bib-0031] Friedman, N. P. , and T. W. Robbins . 2022. “The Role of Prefrontal Cortex in Cognitive Control and Executive Function.” Neuropsychopharmacology 47, no. 1: 72–89. 10.1038/s41386-021-01132-0. 34408280 PMC8617292

[brb370295-bib-0032] Hartwig, F. P. , G. Davey Smith , and J. Bowden . 2017. “Robust Inference in Summary Data Mendelian Randomization via the Zero Modal Pleiotropy Assumption.” International Journal of Epidemiology 46, no. 6: 1985–1998. 10.1093/ije/dyx102. 29040600 PMC5837715

[brb370295-bib-0033] Hemani, G. , J. Zheng , B. Elsworth , et al. 2018. “The MR‐Base Platform Supports Systematic Causal Inference Across the Human Phenome.” eLife 7: e34408. 10.7554/eLife.34408. 29846171 PMC5976434

[brb370295-bib-0034] Holland, G. , V. Jayasekeran, N. Pendleton , M. Horan , M. Jones , and S. Hamdy . 2011. “Prevalence and Symptom Profiling of Oropharyngeal Dysphagia in a Community Dwelling of an Elderly Population: A Self‐Reporting Questionnaire Survey.” Diseases of the Esophagus 24, no. 7: 476–480. 10.1111/j.1442-2050.2011.01182.x. 21385285

[brb370295-bib-0035] Hu, C. , D. Yu , X. Sun , M. Zhang , L. Wang , and H. Qin . 2017. “The Prevalence and Progression of Mild Cognitive Impairment Among Clinic and Community Populations: A Systematic Review and Meta‐Analysis.” International Psychogeriatrics 29, no. 10: 1595–1608. 10.1017/S1041610217000473. 28884657

[brb370295-bib-0036] Jo, S. Y. , J. W. Hwang , and S. B. Pyun . 2017. “Relationship Between Cognitive Function and Dysphagia After Stroke.” Annals of Rehabilitation Medicine 41, no. 4: 564–572. 10.5535/arm.2017.41.4.564. 28971040 PMC5608663

[brb370295-bib-0037] Kertscher, B. , R. Speyer , E. Fong , A. M. Georgiou , and M. Smith . 2015. “Prevalence of Oropharyngeal Dysphagia in the Netherlands: A Telephone Survey.” Dysphagia 30, no. 2: 114–120. 10.1007/s00455-014-9584-z. 25432669

[brb370295-bib-0038] Lawlor, D. A. , R. M. Harbord , J. A. C. Sterne , N. Timpson , and G. Davey Smith . 2008. “Mendelian Randomization: Using Genes as Instruments for Making Causal Inferences in Epidemiology.” Statistics in Medicine 27, no. 8: 1133–1163. 10.1002/sim.3034. 17886233

[brb370295-bib-0039] Leira, J. , A. Maseda , L. Lorenzo‐López , et al. 2023. “Dysphagia and Its Association With Other Health‐Related Risk Factors in Institutionalized Older People: A Systematic Review.” Archives of Gerontology and Geriatrics 110: 104991. 10.1016/j.archger.2023.104991. 36906939

[brb370295-bib-0040] Lewis, C. , M. Walterfang , D. Velakoulis , and A. P. Vogel . 2018. “A Review: Mealtime Difficulties Following Frontotemporal Lobar Degeneration.” Dementia and Geriatric Cognitive Disorders 46, no. 5–6: 285–297. 10.1159/000494210. 30423586

[brb370295-bib-0041] Madan, C. R. , and A. Singhal . 2012. “Motor Imagery and Higher‐Level Cognition: Four Hurdles Before Research Can Sprint Forward.” Cognitive Processing 13, no. 3: 211–229. 10.1007/s10339-012-0438-z. 22466605

[brb370295-bib-0042] Maeda, K. , H. Wakabayashi , H. Shamoto , and J. Akagi . 2018. “Cognitive Impairment Has No Impact on Hospital‐Associated Dysphagia in Aspiration Pneumonia Patients.” Geriatrics & Gerontology International 18, no. 2: 233–239. 10.1111/ggi.13164. 28940784

[brb370295-bib-0043] Malandraki, G. A. , B. P. Sutton , A. L. Perlman , D. C. Karampinos , and C. Conway . 2009. “Neural Activation of Swallowing and Swallowing‐Related Tasks in Healthy Young Adults: An Attempt to Separate the Components of Deglutition.” Human Brain Mapping 30, no. 10: 3209–3226. 10.1002/hbm.20743. 19247994 PMC6870848

[brb370295-bib-0044] Marie, V. , C. Chia Yen , N. Benjamin , and D. Ron . 2018. “Detection of Widespread Horizontal Pleiotropy in Causal Relationships Inferred From Mendelian Randomization Between Complex Traits and Diseases.” Nature Genetics 50, no. 5: 693–698. 10.1038/s41588-018-0099-7. 29686387 PMC6083837

[brb370295-bib-0045] Martin, R. , A. Barr , B. MacIntosh , et al. 2007. “Cerebral Cortical Processing of Swallowing in Older Adults.” Experimental Brain Research 176, no. 1: 12–22. 10.1007/s00221-006-0592-6. 16896984

[brb370295-bib-0046] Michel, A. , E. Vérin , X. Gbaguidi , L. Druesne , F. Roca , and P. Chassagne . 2018. “Oropharyngeal Dysphagia in Community‐Dwelling Older Patients With Dementia: Prevalence and Relationship With Geriatric Parameters.” Journal of the American Medical Directors Association 19, no. 9: 770–774. 10.1016/j.jamda.2018.04.011. 29861192

[brb370295-bib-0047] Moon, H. I. , S. Y. Yoon , T. I. Yi , Y. J. Jeong , and T. H. Cho . 2018. “Lesions Responsible for Delayed Oral Transit Time in Post‐Stroke Dysphagia.” Dysphagia 33, no. 3: 321–328. 10.1007/s00455-017-9856-5. 29022086

[brb370295-bib-0048] Ney, D. M. , J. M. Weiss , A. J. H. Kind , and J. Robbins . 2009. “Senescent Swallowing: Impact, Strategies, and Interventions.” Nutrition in Clinical Practice 24, no. 3: 395–413. 10.1177/0884533609332005. 19483069 PMC2832792

[brb370295-bib-0049] Petersen, R. C. , O. Lopez , M. J. Armstrong , et al. 2018. “Practice Guideline Update Summary: Mild Cognitive Impairment: Report of the Guideline Development, Dissemination, and Implementation Subcommittee of the American Academy of Neurology.” Neurology 90, no. 3: 126–135. 10.1212/WNL.0000000000004826. 29282327 PMC5772157

[brb370295-bib-0050] Pierce, B. L. , and S. Burgess . 2013. “Efficient Design for Mendelian Randomization Studies: Subsample and 2‐Sample Instrumental Variable Estimators.” American Journal of Epidemiology 178, no. 7: 1177–1184. 10.1093/aje/kwt084. 23863760 PMC3783091

[brb370295-bib-0051] Pulvermüller, F. , R. L. Moseley , N. Egorova , Z. Shebani , and V. Boulenger . 2014. “Motor Cognition‐Motor Semantics: Action Perception Theory of Cognition and Communication.” Neuropsychologia 55: 71–84. 10.1016/j.neuropsychologia.2013.12.002. 24333695

[brb370295-bib-0052] Qiao, J. , Z. Wu , X. Cheng , et al. 2022. “Effects of Insular Cortex on Post‐Stroke Dysphagia: A Systematic Review and Meta Analysis.” Brain Sciences 12, no. 10: 1334. 10.3390/brainsci12101334. 36291268 PMC9599629

[brb370295-bib-0053] Roberts, A. , P. Nguyen , J. B. Orange , M. Jog , K. A. Nisbet , and K. McRae . 2017. “Differential Impairments of Upper and Lower Limb Movements Influence Action Verb Processing in Parkinson disease.” Cortex 97: 49–59. 10.1016/j.cortex.2017.09.022. 29080416

[brb370295-bib-0054] Rodrigues, B. , A. C. Nóbrega , M. Sampaio , N. Argolo , and A. Melo . 2011. “Silent Saliva Aspiration in Parkinson's Disease.” Movement Disorders 26, no. 1: 138–141. 10.1002/mds.23301. 21322025

[brb370295-bib-0055] Roldan‐Vasco, S. , A. Orozco‐Duque , J. C. Suarez‐Escudero , and J. R. Orozco‐Arroyave . 2021. “Machine Learning Based Analysis of Speech Dimensions in Functional Oropharyngeal Dysphagia.” Computer Methods and Programs in Biomedicine 208: 106248. 10.1016/j.cmpb.2021.106248. 34260973

[brb370295-bib-0056] Rösler, A. , S. Pfeil , H. Lessmann , J. Höder , A. Befahr , and W. von Renteln‐Kruse . 2015. “Dysphagia in Dementia: Influence of Dementia Severity and Food Texture on the Prevalence of Aspiration and Latency to Swallow in Hospitalized Geriatric Patients.” Journal of the American Medical Directors Association 16, no. 8: 697–701. 10.1016/j.jamda.2015.03.020. 25933727

[brb370295-bib-0057] Saito, T. , K. Hayashi , H. Nakazawa , and T. Ota . 2016. “Clinical Characteristics and Lesions Responsible for Swallowing Hesitation After Acute Cerebral Infarction.” Dysphagia 31, no. 4: 567–573. 10.1007/s00455-016-9716-8. 27277890 PMC4938849

[brb370295-bib-0058] Sakai, K. , H. Hirano , Y. Watanabe , et al. 2016. “An Examination of Factors Related to Aspiration and Silent Aspiration in Older Adults Requiring Long‐Term Care in Rural Japan.” Journal of Oral Rehabilitation 43, no. 2: 103–110. 10.1111/joor.12349. 26432521

[brb370295-bib-0059] Savage, J. E. , P. R. Jansen , S. Stringer , et al. 2018. “Genome‐Wide Association Meta‐Analysis in 269,867 Individuals Identifies New Genetic and Functional Links to Intelligence.” Nature Genetics 50, no. 7: 912–919. 10.1038/s41588-018-0152-6. 29942086 PMC6411041

[brb370295-bib-0060] Seçil, Y. , Ş. Arıcı , T. K. İncesu, N. Gürgör , Y. Beckmann , and C. Ertekin . 2016. “Dysphagia in Alzheimer's Disease.” Neurophysiologie Clinique = Clinical Neurophysiology 46, no. 3: 171–178. 10.1016/j.neucli.2015.12.007. 26924307

[brb370295-bib-0061] Seyedsalehi, A. , V. Warrier , R. A. I. Bethlehem , B. I. Perry , S. Burgess , and G. K. Murray . 2022. “Educational Attainment, Structural Brain Reserve and Alzheimer's Disease: A Mendelian Randomization Analysis.” Brain 146, no. 5: 2059–2074. 10.1093/brain/awac392. PMC10151197 36310536

[brb370295-bib-0062] Simões, A. L. S. , A. Oliva Filho , and E. Hebling . 2020. “Signs for Early Detection of Dysphagia in Older Adults With Severe Alzheimer's Disease.” Journal of Nutrition, Health & Aging 24, no. 6: 659–664. 10.1007/s12603-020-1382-8.32510120

[brb370295-bib-0063] Suh, M. K. , H. Kim , and D. L. Na . 2009. “Dysphagia in Patients With Dementia: Alzheimer Versus Vascular.” Alzheimer Disease and Associated Disorders 23, no. 2: 178–184. 10.1097/WAD.0b013e318192a539. 19474573

[brb370295-bib-0064] Sun, X. , B. Liu, Y. Chen, L. Lv, D. Ye , and Y. Mao . 2022. “Modifiable Risk Factors for Intracranial Aneurysms: Evidence From Genetic Studies.” International Journal of Stroke 17, no. 10: 1107–1113. 10.1177/17474930211065640. 36408635

[brb370295-bib-0065] The 1000 Genomes Project Consortium . 2010. “A Map of Human Genome Variation From Population‐Scale Sequencing.” Nature 467, no. 7319: 1061–1073. 10.1038/nature09534. 20981092 PMC3042601

[brb370295-bib-0066] Thiyagalingam, S. , A. E. Kulinski , B. Thorsteinsdottir , K. L. Shindelar , and P. Y. Takahashi . 2021. “Dysphagia in Older Adults.” Mayo Clinic Proceedings 96, no. 2: 488–497. 10.1016/j.mayocp.2020.08.001. 33549267

[brb370295-bib-0067] van der Maarel‐Wierink, C. D. , J. N. O. Vanobbergen , E. M. Bronkhorst , J. M. G. A. Schols, and C. de Baat . 2011. “Meta‐Analysis of Dysphagia and Aspiration Pneumonia in Frail Elders.” Journal of Dental Research 90, no. 12: 1398–1404. 10.1177/0022034511422909. 21940518

[brb370295-bib-0068] Ward, A. , H. M. Arrighi , S. Michels , and J. M. Cedarbaum . 2012. “Mild Cognitive Impairment: Disparity of Incidence and Prevalence Estimates.” Alzheimer's & Dementia 8, no. 1: 14–21. 10.1016/j.jalz.2011.01.002. 22265588

[brb370295-bib-0069] Yang, H. , K. K. Ang , C. Wang , K. S. Phua , and C. Guan . 2016. “Neural and Cortical Analysis of Swallowing and Detection of Motor Imagery of Swallow for Dysphagia Rehabilitation—A Review.” Progress in Brain Research 228: 185–219. 10.1016/bs.pbr.2016.03.014. 27590970

[brb370295-bib-0070] Yang, E. , K. Kim , J. Lim , and N. Paik . 2014. “Relationship Between Dysphagia and Mild Cognitive Impairment in a Community‐Based Elderly Cohort: The Korean Longitudinal Study on Health and Aging.” Journal of the American Geriatrics Society 62, no. 1: 40–46. 10.1111/jgs.12606. 25180377

[brb370295-bib-0071] Yatabe, N. , K. Takeuchi , M. Izumi , et al. 2018. “Decreased Cognitive Function Is Associated With Dysphagia Risk in Nursing Home Older Residents.” Gerodontology 35, no. 4: 376–381. 10.1111/ger.12366. 30028036

[brb370295-bib-0072] Yokota, J. , Y. Ogawa , S. Yamanaka , et al. 2016. “Cognitive Dysfunction and Malnutrition Are Independent Predictor of Dysphagia in Patients With Acute Exacerbation of Congestive Heart Failure.” PLoS ONE 11, no. 11: e0167326. 10.1371/journal.pone.0167326. 27898735 PMC5215957

[brb370295-bib-0073] Zhao, Q. , J. Wang , G. Hemani, J. Bowden , and D. S. Small . 2019. “Statistical Inference in Two‐Sample Summary‐Data Mendelian Randomization Using Robust Adjusted Profile Score.” arXiv. 10.48550/arXiv.1801.09652.

